# Biochemical and microbiological activity of soil contaminated with *o*-cresol and biostimulated with *Perna canaliculus* mussel meal

**DOI:** 10.1007/s10661-018-6979-6

**Published:** 2018-09-21

**Authors:** Magdalena Zaborowska, Jan Kucharski, Jadwiga Wyszkowska

**Affiliations:** University of Warmia and Mazury in Olsztyn, Plac Łódzki 3, 10-727 Olsztyn, Poland

**Keywords:** *o*-Cresol, Soil, Oxidoreductases, Hydrolases, Microorganisms, Mussel

## Abstract

The choice of the study subject was a consequence of the growing interest in volatile organic compounds which are strongly dispersed in the environment. The knowledge of *o*-cresol’s capability for being broken down by bacteria should be supplemented by studies aimed at determining the biochemical and microbiological activity of soils. *o*-Cresol was applied at the following rates: 0, 0.1, 1, 10, and 50 mg of *o*-cresol kg^−1^ d.m. of soil to determine its effect on the biological properties of soil. The activity of dehydrogenases, catalase, urease, acid phosphatase, alkaline phosphatase, arylsulfatase, and *β*-glucosidase, the eight groups of microorganism counts, was determined in soil samples after 45 days and the barley yield was determined. Preventive biostimulation with *Perna canaliculus* mussel meal, illustrated by means of the index of fertility (IF), was conducted in order to eliminate the adverse effect of *o*-cresol. The soil and crop resistance index (RS) was used to illustrate the response of barley, and R:S—the rhizosphere effect index was used to determine the effect of the crop on the enzymatic activity of soil. *o*-Cresol had a beneficial effect on the biological activity of soil at an acceptable rate of 0.1 and 1 mg kg^−1^ d.m. of soil, and it became its inhibitor after being applied at 10 and 50 mg kg^−1^ d.m. of soil, which also brought about a decrease in the resistance of spring barley. Dehydrogenases are the most sensitive, and catalase is the least sensitive, to the pressure of *o*-cresol in soil. Mussel meal can be recommended as a biostimulator of soil fertility. It also eliminated the negative effect of *o*-cresol on its biological activity.

## Introduction

Cresol is a volatile organic compound of the phenols group. It has a molecular weight of 108.14 mg mol^−1^ (OECD 2005) and occurs as three structural isomers, one of them being *o*-cresol with the methyl group in the ortho position (Mikami et al. 2016; Badanthadka and Mehendale 2014). Synonyms of 2-methylphenol: 2-hydroxytoluen and *o*-cresylic acid. Owing to a large pool of anthropogenic and natural sources of *o*-cresol in the environment, this compound can be included in the ATSDR’s Substances Priority List (ATSDR 2017). The prime objective of this concept is to present substances based on a combination of their occurrence and toxicity.

Natural sources of *o*-cresol include fruit, vegetables, tea, and cocoa (Bronick and Lal 2005). It is also formed by biodegradation of lignins and tannins (Kaisoon et al. 2011). On the other hand, anthropogenic environmental pollution is a consequence of the development of the chemical, paper, and textile industry (Marrot et al. 2006; Arutchelvan et al. 2005). It is worrying that *o*-cresol is present in herbicides. One example is 4,6-dinitro-*o*-cresol, which is a component of insecticides and acaricides, still distributed in third countries, with a high mobility in soil, undergoing slow microbiological degradation in soil (Uzer et al. 2006). *o*-Cresol has also been found in coal tar, in crude oil, and in fly ash from burning wood (Badanthadka and Mehendale 2014). The problem is aggravated by the use of cresol as a frother in metallurgical flotation in mining. Being a surfactant, it is adsorbed on the water-air boundary (Vecino et al. 2013). Its global annual production output amounts to 3 Mg (Zhu et al. 2018). This policy will result in increasing soil degradation caused by releasing growing amounts of toxic binary compounds of copper and cresol (Nguyen et al. 2013).

To understand better the complexity of interactions between methylphenols with elements of soil ecosystems, a comprehensive approach must be considered also based on the influence and features of plants—both of their aboveground parts and roots (Steinauer et al. 2017). According to Diaz et al. (2016), it is the functions of plants that are used to predict the distribution of biodiversity. This holistic vision of the environment assessment fits conveniently into the subject matter of phenols, including *o*-cresol, since plants are natural sources of a great number of phenolic secondary metabolites. They change their polarity, volatility, chemical stability in cells, and, in consequence, biological activity (Cheynier et al. 2013). Polyphenolic compounds which contain flavonoids and phenolic acids can be divided into benzoic acid derivatives (gallic acid, protocatechuic acid), derivatives of cinnamic acid (coumaric, caffeic and ferulic acids) and stilbenes (Daglia 2012). Flavan-3-ols, flavonols, and tannins are polyphenols capable of suppressing many virulence factors of microorganisms, including inhibition of biofilm formation and neutralization of bacterial toxins (Vaquero et al. 2010). The structure and activity of the soil microbiome are modulated in a particular manner by plants, mainly through root secretions, such as phytohormones, salicylic acid (Lebeis et al. 2015), or the brachialactone—a nitrification inhibitor (Subbaro et al. 2009). Approximately 5–25% of carbon in plants is released with their secretions to soil (Derrien et al. 2004) where it is usually rapidly transformed by microorganisms (Paterson et al. 2011).

Cresols can be used preferentially as a source of carbon and energy for microorganisms (Ren et al. 2014). However, hydroxyl groups equivalent to the presence of molecular oxygen as a co-substrate must then occur in the environment (Harwood et al. 1999). According to Patil and Anil (2015), the exposure of microorganisms to cresols leads to changes in the permeability of the cell membrane of microorganisms causing its depolarization, which ultimately contributes to the disappearance of the energy difference between the cell’s exterior and interior and it is equivalent to its autolysis and death.

To date, surprisingly few attempts have been made to assess the effect of cresols on soil biochemical activity (Zhu et al. 2018). This triggers the need for such studies, arguing that it has been reported that this group of compounds has a toxic effect on the human body. According to Sanders et al. (2009), cresol contributes to an increase in the incidence of renal tubular adenoma. Finally, in the human body, cresols are conjugated with glucuronic acid and sulfonated and then excreted in urine (Morinaga et al. 2004).

Enzymatic activity is a component of soil process simulation models (Schimel et al. 2017) and considered to be a critical parameter (Manzoni et al. 2016). They may be denatured by excessive heat, react with minerals, be absorbed intact by microorganisms, and be metabolized in cells (Burns et al. 2013). Enzyme activity indirectly affects the soil’s ability to degrade contaminants (Kucharski et al. 2016; Wyszkowska et al. 2017; Schimel et al. 2017). It is not without reason that specific enzymes are selected for studies, as they are the basis for determination of fertility indices. The BA = DEH + CAT + PAL + PAC + URE + GLU + ARYL, consisting of seven enzymes: dehydrogenases (DEH), acid phosphatase (PAC), alkaline phosphatase (PAL), urease (URE), catalase (CAT), *β*-glucosidase (GLU) and arylsulfatase (ARYL) is considered highly reliable (Borowik et al. 2017; Wyszkowska et al. 2013).

The preventive measures aimed to eliminate the potential inhibitory effects of *o*-cresol were an important step in the research. Meal made from *Perna canaliculus* mussels from New Zealand, regarded as a sentinel species for soils contaminated with heavy metals, was applied to the soil (Chandurvelan et al. 2013). Three main metabolites of the mussels, which make it an effective biostimulator, include peptides, lipids containing polyunsaturated fatty acids (PUFA), and polysaccharides (Grienke et al. 2014).

The knowledge of diverse degradation activity of microbial activity towards cresols is increasing, but it is not parallel to the knowledge of the effect of increasing pollution of soil with 2-methylphenols on its biochemical activity, which provides the reason for this study. Therefore, the prime objective of this study is to determine the effect of *o*-cresol on the activity of soil enzymes and microorganisms exposed to the pressure of increasing doses of this phenolic compound in soil.

## Materials and methods

### Experimental: soil sampling and sample preparation

The appropriate soil was taken from the arable-humus horizon of brown soil (Eutric Cambisol) at the Teaching and Research Centre in Tomaszkowo (NE Poland, 53.7161° N, 20.4167° E). The results of granulometric analysis and selected physical and chemical properties are shown in Table 1. It was a soil of the granulometric composition of loamy sand (IUSS Working Group WRB 2014). The physical and chemical properties of the soil, which included pH in 1 mol dm^−3^ KCl, hydrolytic acidity, total organic carbon content, and sum of exchangeable cations (K^+^, Na^+^, Ca^2+^, Mg ^2+^), were determined by the methods described in the paper by Borowik et al. (2017).

**Table 1:** Some physicochemical properties of the soil used in the experiment

Properties	Unit	Value
Granulometric composition of soil (percentage of fraction (*d*))	2.00 ≥ *d* ≥ 0.05 mm	69.41
	0.05 ≥ *d* > 0.002 mm	27.71
	*d* ≤ 0.002 mm	2.88
pH_KCl_		7.0
HAC	mM (+) kg^−1^ d.m. of soil	6.40
EBC		165.90
CEC		172.30
BS	(%)	96.29
Corg	g kg^−1^ d.m. of soil	6.40
K_e_		180.00
Ca_e_	mg kg^−1^ d.m. of soil	2571.40
Na_e_		20.00
Mg_e_		59.50

*HAC*, hydrolytic acidity; *EBC*, sum of exchangeable base cations; *CEC*, cation exchange capacity; *BS*, base saturation; *pH*_*KCl*_, soil reaction; *e*, exchangeable

The experiment was carried out in the vegetation hall. The main variable factors were: (1) the dose of *o*-cresol: 0; 0.1; 1; 10 and 50 mg of *o*-cresol kg^−1^ d.m. of soil, (2) the addition of the biostimulator *Perna canaliculus* mussel meal: 0; 5 mg kg^−1^ d.m. of soil, and (3) sowing spring barley cv. *Conchita* in the soil. *o*-Cresol of confirmed 99% purity was purchased from Sigma-Aldrich.

Properly prepared soil was used to set up a pot experiment in five replications. To this end, soil contaminated with *o*-cresol was mixed in polyethylene containers with NPKMg fertilizers. Mineral fertilization rates, converted into pure elements and expressed in mg kg^−1^, were as follows: N – 250; P – 50; K – 90; Mg - 20, Cu – 5; Zn – 5; Mo – 5; Mn – 5 and B – 0.33. Nitrogen was applied as urea, phosphorus, and potassium as potassium dihydrogen phosphate, magnesium as magnesium sulfate heptahydrate, copper as cooper(II) sulfate pentahydrate, zinc as zinc chloride, molybdenum as sodium molybdate dihydrate, manganese as manganese(II) chloride tetrahydrate, and boron as boric acid. Meal from New Zealand *Perna canaliculus* mussel was added to soil in selected pots. The content of nitrate and ammonium nitrogen in this fertilizing agent was N–NO_3_, 4.98 mg kg^−1^ d.m, and N–NH_4_, 1902.25 mg kg^−1^ d.m. After mixing it with *o*-cresol and the fertilizers, 1 kg of the soil was put into each of 1.5 dm^3^ pots and its moisture content was brought to 60% capillary water capacity. This parameter was monitored throughout the experiment and kept at a constant level. Vegetation of spring barley cv. *Conchita* was conducted for 45 days. After germination, five plants were left in each pot. The dry yield of the plant and roots was determined after harvesting the barley at the BBCH 52 phase-heading (20% of inflorescence emerged).

### Microbiological and biochemical analysis

On day 45 of the study, the activity of seven soil enzymes, except catalase (EC 1.11.1.6), was determined with a Perkin-Elmer Lambda 25 spectrophotometer (Ma, USA). The description of substrates and units of activity of the various enzymes was characterized: dehydrogenases (EC 1.1) determined at wavelength (*λ*) 485 nm, urease (EC 3.5.1.5), acid phosphatase (EC 3.1.3.2) and alkaline phosphatase (EC 3.1.3.1) at 410 nm arylsulfatases (EC 3.1.6.1) at 420 nm by Zaborowska et al. (2016) and *β*-glucosidase (EC 3.2.1.21) measured at 400 nm by Borowik et al. (2017).

In the same soil samples, populations of eight groups of microorganisms: organotrophic ammonifying bacteria, nitrogen fixing bacteria, *Arthrobacter* sp. and *Pseudomonas* sp., *Azotobacter* sp., *Actinobacteria* and fungi were determined. The microorganisms count was determined with a colony counter. The microbial media used in the experiment are characterized in Borowik et al. (2017).

### Calculations and statistical analysis

Statistical analyses were conducted using Statistica 10.0 software (StatSoft inc. 2018). The *η*^2^ coefficient of percentile variability of the variable under study was determined by analysis of variance—ANOVA. A multi-dimensional and exploratory analysis principal components analysis (PCA) was conducted to illustrate the response of each enzyme to soil contamination with *o*-cresol and sowing spring barley on it. An effect of the plant was shown with the rhizosphere effect index R:S. It is the ratio of enzymatic activity in soil sown with spring barley (R) and in unsown soil (S). Changes of the activity of the eight groups of microorganisms were monitored by the cluster method—Ward’s dendrogram. The colony development (CD) index (Sarathchandra et al. 1997) and the ecophysiological diversity (EP) (De Leij et al. 1993) were determined for the organotrophic bacteria, *Actinobacteria*, and fungi counted for 10 successive days.

The index of an effect of a fertilizing agent—IF—described in Zaborowska et al. (2017) was used to make an assessment of the effect of the mussel meal on the microbiological and biochemical activity of soil. Homogeneous variance between groups of microorganisms was determined with Tukey’s test at *P* = 0.01. Furthermore, the barley yield was used to determine the plant resistance to contamination of soil with *o*-cresol. The calculations were based on the formula presented in Orwin and Wardle (2004). An index (PR) has been proposed, which is the ratio of the dry yield of the aboveground parts of a crop (P) and the dry yield of its roots (R), to emphasize the response of roots to a growing pressure of *o*-cresol in soil.

## Result and discussion

The research presents a comprehensive characterization of the effect of *o*-cresol on biochemical and microbiological activity, as well as the response of spring barley to the pressure of this phenolic compound. High values of the *η*^2^ coefficient indicate that enzyme activity was moderated by increasing doses of *o*-cresol to a greater extent than soil microbiome (Table 2). The highest values of *η*^2^ were recorded for dehydrogenases (85.64%) and for fungi (68.79%).

**Table 2:** The percentage of the observed variability determined by the value of coefficient *η*^2^

The variable	Enzyme activity								Average
	DEH	URE	PAC	PAL	CAT	ARYL	GLU		
D	85.640	13.100	12.570	1.018	0.582	16.216	45.170		24.899
S	3.414	69.029	7.694	97.830	96.336	60.644	0.047		47.856
D·S	8.061	17.215	78.042	0.661	2.867	21.870	41.005		24.246
Error	2.886	0.656	1.695	0.491	0.215	1.269	13.778		2.998
	Number of microorganisms								
	Arth	Ps	Az	Im	Am	Org	Act	Fun	
D	9.948	4.381	26.590	17.630	0.971	8.518	5.933	68.790	17.845
S	80.769	88.828	47.375	81.700	96.201	74.071	71.816	2.832	67.949
D·S	6.859	5.117	13.504	0.525	2.609	14.868	17.541	24.289	10.664
Error	2.424	1.675	12.531	0.145	0.219	2.542	4.710	4.088	3.542

*D*, dose; *S*, mussels of *Perna canaliculus*; DEH, dehydrogenases; URE, urease; PAC, acid phosphatase; PAL, alkaline phosphatase; CAT, catalase; GLU, *β*-glucosidase; ARYL, arylsulfatase; Ps, *Pseudomonas* sp.; Arth, *Arthrobacter* sp.; Az, *Azotobacter* sp*.*; Am, ammonifying bacteria; Im, immobilizing bacteria; Org, organotrophic bacteria; Act, *Actinobacteria*; Fun, fungi

**Fig. 1:**
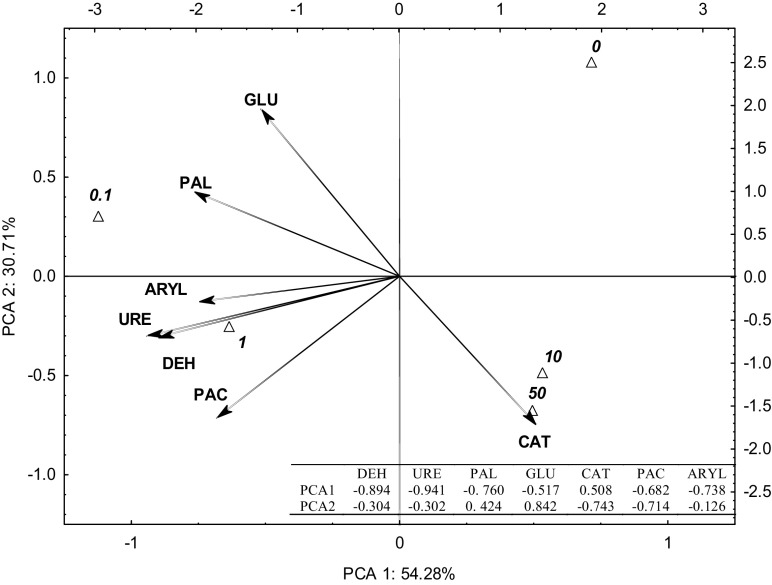
Enzyme activity in soil contaminated with *o*-cresol—PCA method. Vectors represent the analyzed variables: DEH, dehydrogenases; CAT, catalase; URE, urease; PAL, alkaline phosphatase; PAC, acid phosphatase; GLU, *β*-glucosidase; ARYL, arylsulfatase—0, 0.1, 1, 10, and 50 doses of *o*-cresol in mg kg^−1^ d.m. of soil

The PCA provided a detailed insight into the sensitivity of each enzyme to the presence of *o*-cresol in soil (Fig. 1). The distribution of vectors around the axis representing the first factor (which determines 54.28% of the total data variance) indicates that the activity of acid phosphatase (PAC), arylsulfatase (ARYL), urease (URE), dehydrogenase (DEH), *β*-glucosidase (GLU), and alkaline phosphatase (PAL) was correlated negatively with the variable. A positive correlation was observed only in the activity of catalase (CAT) which described the second factor, which characterized 30.71% of the variability of results. Coordinates of cases and distances between them show that the enzymatic activity was higher in pots where soil was contaminated with *o*-cresol at 0.1 and 1 mg kg^−1^ d.m. of soil than in the control sample. However, the activity of all of the enzymes except catalase was inhibited following the addition of 10 and 50 mg of *o*-cresol kg^−1^ d.m. of soil. *o*-Cresol proved to have significant inhibitory properties towards GLU and PAL, which corresponds to the lowest correlation coefficients for these enzymes: *r* = (− 0.846) and *r* = (− 0.595), respectively. A dose of 0.1 mg of *o*-cresol kg^−1^ d.m. of soil stimulated the activity of GLU and PAL, whereas the application of 1 mg of *o*-cresol kg^−1^ d.m. of soil boosted the activity of DEH > URE > ARYL = PAC, for which the ultimate correlation coefficients were *r* = − 0.391, *r* = − 0.285, *r* = 0.165, and *r* = 0.169, respectively. Furthermore, the activity of catalase was significantly positively correlated with increasing contamination of soil with *o*-cresol (*r* = 0.611).

According to Guangming et al. (2017), the activity of alkaline phosphatase, urease, and catalase is closely correlated with organic matter in soil. Therefore, the addition of *o*-cresol (0.1–1 mg kg^−1^ d.m. of soil) became a source of carbon and energy stimulating the activity of majority of enzymes. However, as in this study, Zhu et al. (2018) observed decreased activity of urease. It may be caused by the presence in soil of catechol, which is an indirect metabolite of *o*-cresol (Ren et al. 2014) and a strong inhibitor of urease, deactivating it according to the mechanism associated with the thiol group of α Cys 322 (Mazzei et al. 2016, 2017). One of the functions of catalase in soil is to decompose the hydrogen peroxide formed in metabolic processes, which prevents its toxic effect on living organisms (Guangming et al. 2017). A higher activity was exhibited by catalases, which contain heme and an iron-porphyrin cofactor (Grigoras 2017).

**Fig. 2:**
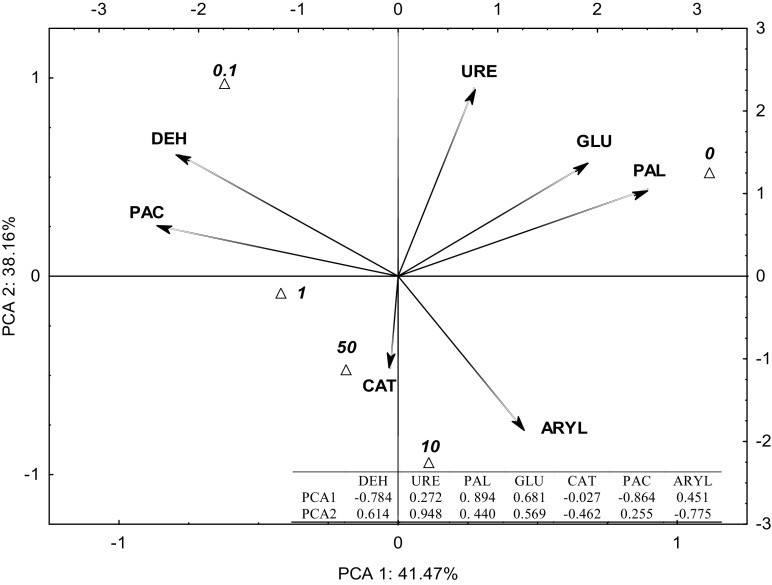
The rhizosphere effect (R:S) enzyme activity in soil contaminated with *o*-cresol—PCA method (for abbreviations, see Fig. 1)

The effect of the crop on the biochemical activity of soil was also analyzed to make the experiment more significant. To this end, the rhizosphere effect index (R:S) for the activity of individual soil enzymes was used (Fig. 2). The PCA analysis emphasized the observed trends. Vectors corresponding to R:S for GLU, PAL, and DEH were distributed around the axis which represented the first explaining variable for 41.47% of the total data variance. A second variable for 38.16% of data variance contained vectors related to the values of R:S for URE, ARYL, CAT, and PAC. The values of vectors representing the R:S index, affected by the first principle component (PCA 1), were negative for DEH (− 0.784) and PAC (− 0.864), and positive for the other enzymes, ranging from 0.272 for URE to 0.894 for PAL. The second principal component (PCA 2) generated positive values of vectors for all enzymes except CAT and ARYL. Barley was found to have a beneficial effect on the activity of URE, GLU, and PAL in control samples, but the opposite trend was observed in pots with the largest dose of 50 mg of *o*-cresol kg^−1^ d.m. of soil. The activity of acid phosphatase and dehydrogenases was stimulated to the greatest extent by a combination of spring barley growing in the soil and the application of 0.1–1 mg of *o*-cresol kg^−1^ d.m. of soil. Adding *o*-cresol to the soil at doses which are 100 and 500 times higher than acceptable proved to stimulate the activity of CAT and ARYL to the greatest extent, which is indicated by the coordinates of cases and distances between them.

These findings are confirmed by the report of Xiao et al. (2007), who found isoflavone polyphenols to inhibit the activity of urease due to their structure with two hydroxyl groups in ortho positions. The inhibitory strength of polyphenols does not decrease until after the C-isoflavone ring is broken down. Takishima et al. (1988) claim that SH groups in cysteinyl, which are components of sulfhydryl groups, transformed into cysteine SS bonds by oxidative dehydrogenation of cresols, which decrease the activity of urease. Simultaneous growing of barley in soil and contaminating it with *o*-cresol probably augmented this inhibitory effect.

**Fig. 3:**
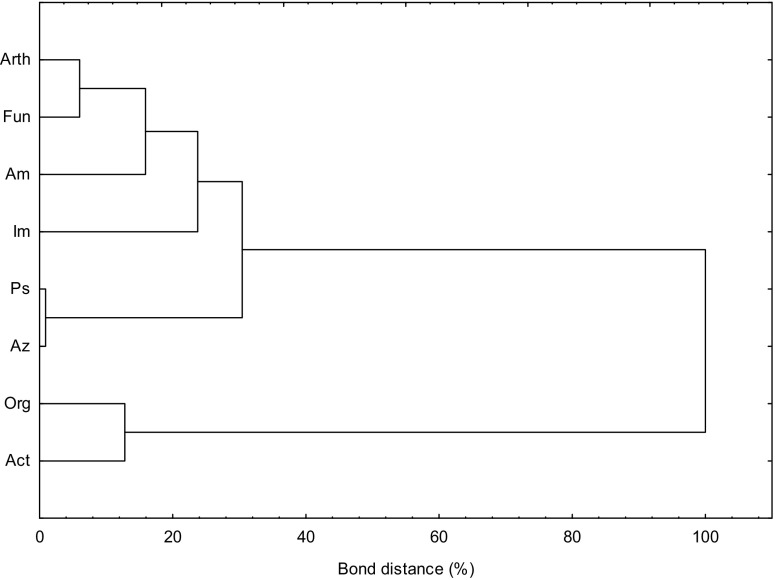
Similarity of microbial reaction to contamination of soil with *o*-cresol. *Arth*, *Arthrobacter* sp.; *Fun*, fungi; *Am*, ammonifying bacteria; *Im*, nitrogen immobilizing bacteria; *Ps*, *Pseudomonas* sp.; *Az*, *Azotobacter* sp.; *Org*, organotrophic bacteria; *Act*, Actinobacteria

The assessment of the disturbance of homeostasis of the soil subjected to pressure of *o*-cresol was supplemented with an analysis of the response of microorganisms to its increasing doses. It was conducted by Ward’s cluster method (Fig. 3). Five subclusters of homogeneous variances which make up one of two principal clusters were formed by fixing bacteria, ammonifying bacteria, and *Arthrobacter*, *Pseudomonas*, and *Azotobacter*. A separate cluster was made up by organotrophic bacteria and *Actinobacteria*.

**Fig. 4:**
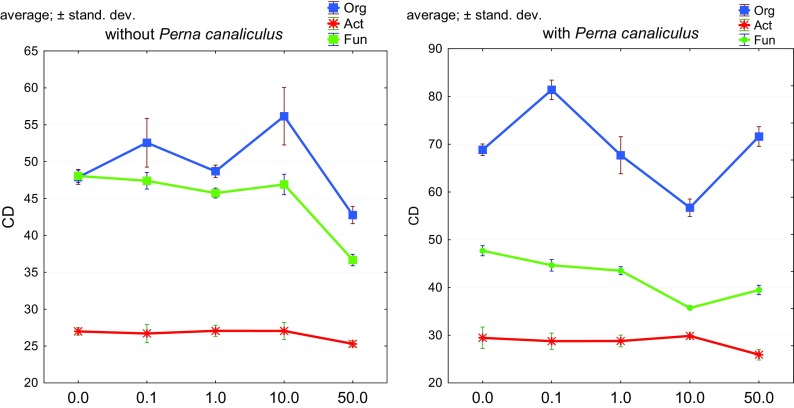
Colony development index (CD) for Org, organotrophic bacteria; Act, *Actinobacteria*; Fun, fungi to soil pollution with *o*-cresol and biostimulation with *Perna canaliculus* mussel meal: 0, 0.1, 1, 10, 50 doses of *o*-cresol (mg *o*-cresol kg^−1^ d.m. of soil)

**Fig. 5:**
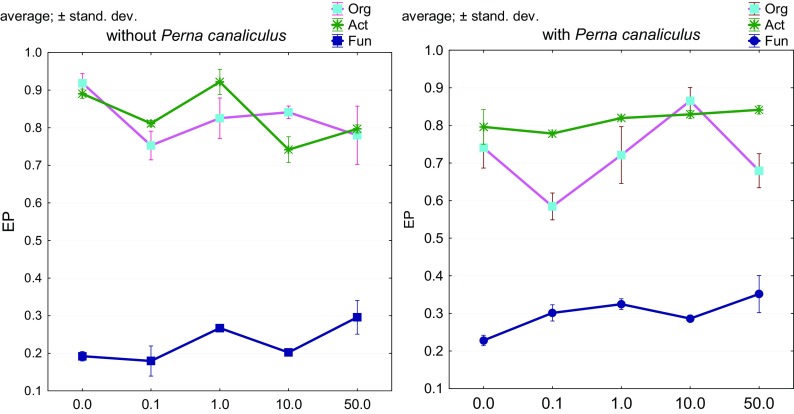
Ecophysiological factor diversity (EP) for Org, organotrophic bacteria; Act, *Actinobacteria*; Fun, fungi to soil pollution with *o*-cresol and biostimulation with *Perna canaliculus* mussel meal (for abbreviations, see Fig. 4)

Valuable information on the complexity of the effect of *o*-cresol on bacteria was provided as a result of the determination of colony development indices (Fig. 4) and indices of ecophysiological diversity (EP) (Fig. 5). These indices emphasized the range of effect that *o*-cresol had on multiplication and changes of the microorganism composition. *o*-Cresol had a beneficial effect on development of organotrophic bacteria and fungi and less so on the development of *Actinobacteria*. Interestingly, organotrophic bacteria multiplied with the greatest intensity in the presence of 10 mg of *o*-cresol kg^−1^ d.m. of soil. An interesting phenomenon is, on the one hand, that an increase in the biodiversity of organotrophic bacteria and *Actinobacteria* was more significant than of fungi and, on the other, that the CD index does not increase after an increased dose of *o*-cresol is applied (50 mg of *o*-cresol kg^−1^ d.m. of soil). It is also surprising that the acceptable dose of the phenolic compound under study—0.1 mg of *o*-cresol kg^−1^ d.m. of soil—had such an inhibitory effect and decreased the EP index for organotrophic bacteria by 21% relative to the control sample.

According to Zhu et al. (2018), although *o*-cresol is less toxic to microorganisms than *m*-cresol, it is a source of carbon and energy for them, which has been observed in this study, even at a dose of 10 mg of *o*-cresol kg^−1^ d.m. of soil. However, it effectively moderates the diversity of microorganisms at this dose. It sorts them and leaves active *Proteobacteria*, including *Pseudomonas* sp., *Arthrobacter* sp., and *Azotobacter* sp. as well as *Acidobacteria* and *Bacteroidetes*. Gram-negative species are dominating microorganisms (Zhu et al. 2018). Patil and Anil (2015) attribute it to a thinner cell wall and a higher isoelectric point (pH = 4–5) in this group of microorganisms. Kristanti et al. (2016) report that the strain of *Absidia spinosa* M15 produces laccase, owing to which it breaks down cresol within 30 days. Atagana (2004) isolated fungi of genera *Aspergillus*, *Candida*, *Cladosporium*, *Fusarium*, *Monicillum*, *Trichoderma*, and *Penicillium* from soil polluted with cresol*.*

It is beyond doubt that another, equally important, factor under study was a potential biostimulator of biological activity of soil—New Zealand *Perna canaliculus* mussel meal*.* This fertilizing substance generated the highest values of CD for organotrophic bacteria in soil with an addition of 0.1 mg of *o*-cresol kg^−1^ d.m. of soil, which resulted in intense colony development in this group compared to soil with no mussel meal added (Fig. 4). It is noteworthy that there was a significant positive correlation between EP for fungi and increasing doses of *o*-cresol (Fig. 5).

The effectiveness of mussel meal in stimulating the biochemical and microbiological activity of soil was analyzed using the IF index (Table 3). It turned out that the substance, as expected, acted as a biostimulator, improving the fertility of the soil significantly. Particularly, high IF was calculated for CAT, URE, and ARYL. On the one hand, the response from CAT should be regarded as unprecedented, because the IF index for this enzyme was the highest in soil with 0.1 mg of *o*-cresol kg^−1^ d.m. of soil (IF = 17.932); on the other, it is similar with DEH, because their activity was slightly stimulated only by a combination of mussel meal and 50 mg of *o*-cresol kg^−1^ d.m. of soil. Considering the mean IF index for enzymes, reflecting their response to fertilization of soil with mussel meal, they can be put in the following sequence: GLU > DEH > PAC > PAL > ARYL > URE > CAT. When this relationship is analyzed through the correlation coefficients, which concern the interactions between increasing doses of *o*-cresol and the fertilizing substance applied, the following sequence should be proposed: ARYL > CAT > URE > PAC > GLU > DEH > PAL.

**Table 3:** Coefficients of impact (IF) of an alleviating substance for enzyme activity and number of microorganisms in soil pollution with *o*-cresol

Dose of *o*-cresol mg kg^−1^ d.m. of soil	Enzyme activity							
	DEH	URE	PAC	PAL	CAT	ARYL	GLU	
0	0.921^b^	6.291^a^	1.465^a^	1.701^a^	10.001^b^	2.654^a^	0.847^b^	
0.1	0.778^c^	2.785^b^	0.961^b^	1.615^b^	17.932^a^	2.144^a^	0.826^b^	
1	0.910^b^	2.375^b^	0.934^b^	1.684^b^	4.809^c^	2.498^a^	1.028^a^	
10	0.942^b^	2.826^b^	0.981^b^	1.655^b^	4.644^c^	2.353^a^	1.066^a^	
50	1.070^a^	1.903^b^	0.955^b^	1.815^b^	3.299^d^	0.970^b^	1.076^a^	
Average	0.924^z^	3.236^w^	1.059^z^	1.694^y^	8.137^u^	2.124^x^	0.969^z^	
*r*	0.810*	− 0.496*	− 0.310	0.802*	− 0.536*	− 0.929*	0.607*	
	Number of microorganisms							
	Am	Im	Arth	Ps	Org	Fun	Act	Az
0	1.248^b^	3.372^b^	5.025^a^	4.608^a^	1.241^c^	1.004^c^	1.324^b^	0.312^b^
0.1	1.514^ab^	3.816^a^	4.331^ab^	3.783^ab^	1.881^b^	1.084^c^	2.655^a^	1.778^a^
1	1.876^ab^	3.502^b^	5.039^a^	3.222^b^	1.984^b^	1.463^b^	2.869^a^	0.527^b^
10	2.006^a^	3.373^b^	4.665^a^	2.858^b^	2.670^a^	0.902^c^	1.610^b^	0.279^b^
50	1.886^ab^	1.832^c^	3.140^b^	5.113^a^	2.799^a^	3.177^a^	1.896^b^	0.273^b^
Average	1.706^xy^	3.179^w^	4.440^t^	3.917^u^	2.115^x^	1.526^y^	2.071^x^	0.634^z^
*r*	0.323	− 0.975*	− 0.855*	− 0.765*	0.724*	0.934*	0.944*	− 0.387

Homogeneous groups specified in columns, for each enzyme and group of microorganisms, depending on the increasing doses of *o*-cresol. *r*, the correlation coefficient. *Significant for *P* = 0.05, *n* = 14

The highest values of IF among all the groups of microorganisms under study were found for *Arthrobacter* (mean IF = 4.440) and *Pseudomonas* (mean IF = 3.917) (Table 3). An addition of the mussel meal had a beneficial effect on the fixing bacteria count. It also stimulated the multiplication of organotrophic bacteria and *Actinobacteria*. The mean values of their indices made up a group of homogeneous variances. A beneficial effect for *Azotobacter* sp. was observed when the soil was fertilized with the mussel meal only in combination with the smallest dose of *o*-cresol.

The mussel meal performed its intended function and improved soil fertility due to the fact that the New Zealand mussel *Perna canaliculus* is a source of protein, lipids, and carbohydrate (Grienke et al. 2014), which produced a positive response of soil fungi. Moreover, Srisunont and Babel (2015) report that the mussel *Perna viridis* takes up nutrients, and the process efficiency is 61% for carbon, 62% for nitrogen, and 79% for phosphorus. It also excretes these elements to the environment in which it lives, in the following amounts: 108, 35.5, and 46 mg day^−1^, respectively, which makes it potentially a reliable and valuable source of essential elements for microorganisms.

**Fig. 6:**
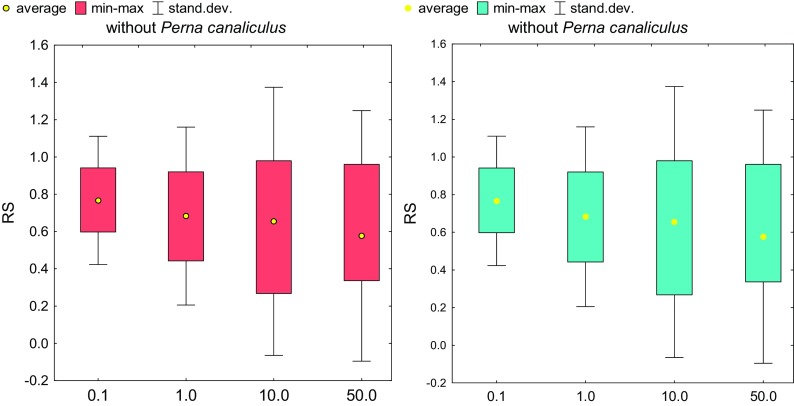
Index of spring barley resistance (RS) depending on *o*-cresol pollution and biostimulation with *Perna canaliculus* mussel meal (for abbreviations, see Fig. 4)

**Fig. 7:**
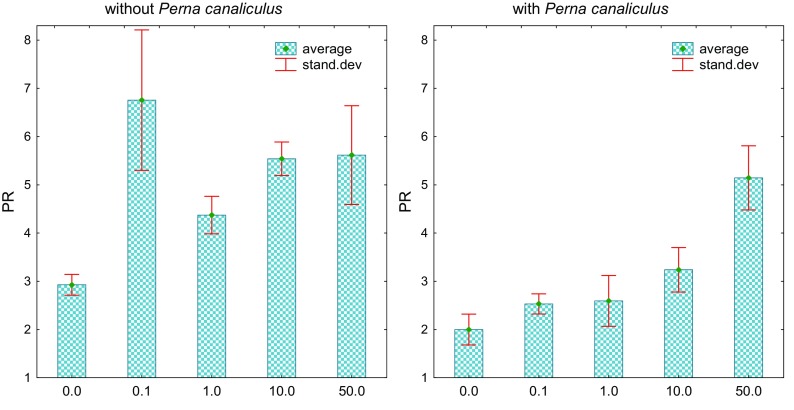
The ratio of dry weight of the aboveground parts of barley (P) to dry weight of the root (R) to soil pollution with *o*-cresol and biostimulation with *Perna canaliculus* mussel meal (for abbreviations, see Fig. 4)

In order to transpose the effect of activity of soil communities to the ecosystem scale, one must assess in detail the changes that take place in it. An important part of the research was to determine the effect of *o*-cresol on spring barley yield, especially since barley itself is a source of phenolic compounds, mainly ferulic acid (Zhu et al. 2015). The plant resistance was found to decrease with increasing soil contamination with *o*-cresol, regardless of whether it had been fertilized with mussel meal or not (Fig. 6). Ferlian et al. (2017) suggest that the quantitative and qualitative characteristics not only of the aboveground parts but also of the roots of the plants should be determined in order to check whether they are coordinated with each other. Using this suggestion, it was shown (Fig. 7) that contamination of soil with *o*-cresol also reduced the weight of the root, which manifested itself in high values of PR, whose strange escalation was observed following the application of the lowest dose of 0.1 mg of *o*-cresol kg^−1^ d.m. of soil. It should be emphasized that the mussel generated nearly 3.5 times lower PR in soil with the acceptable dose and twice lower when 1–10 mg of *o*-cresol kg^−1^ d.m. of soil was added. Its effectiveness decreased considerably under pressure of *o*-cresol at the highest dose of 50 mg of *o*-cresol kg^−1^ d.m. of soil.

## Conclusion

*o*-Cresol significantly moderates the biochemical and microbiological activity of soil. It is a source of carbon and energy essential to soil microbiome at an acceptable dose of 0.1 and 1 mg of *o*-cresol kg^−1^ d.m. of soil. However, at doses exceeding 10 mg of *o*-cresol kg^−1^ d.m. of soil, it inhibited the enzymatic activity of the soil, except CAT, which decreased the resistance of spring barley. Considering the sensitivity of enzymes to the pressure of *o*-cresol, they can be arranged in the following sequence: CAT > PAC = ARYL > URE > DEH > PAL > GLU. The highest doses of *o*-cresol stimulated the multiplication of both organotrophic bacteria and *Actinobacteria*, but they had an adverse effect on their biodiversity. *Perna canaliculus* mussel meal has proven to be a very good biostimulator for both biochemical activity—except dehydrogenases—and microbiological activity, without taking into account the response of *Azotobacter* sp. to the organic substance under test. It also contributed to an increase in the biodiversity of fungi, which increased with the contamination of the soil with *o*-cresol. Soil fertilization with mussel meal alleviated the toxic effects of *o*-cresol on its biological activity. No increase in spring barley yield was observed after the application of mussel meal; however, a much higher root mass was obtained in the pots where it had been added.

## References

[CR1] Arutchelvan V, Kanakasabai V, Nagarajan S, Muralikrishnan V (2005). Isolation and identification of novel high strength phenol degrading bacterial strains from phenol - formaldehyde resin manufacturing industrial wastewater. Journal of Hazardous Materials B.

[CR2] Atagana HI (2004). Biodegradation of phenol, *o*-cresol, *m*-cresol and *p*-cresol by indigenous soil fungi in soil contaminated with creosote. World Journal of Microbiology and Biotechnology.

[CR3] ATSDR (2017). Substance priority list. Atlanta: Agency for Toxic Substances and Disease Registry. http://www.atsdr.cdc.gov/spl/.

[CR4] Badanthadka M., Mehendale H.M. (2014). Cresols. Encyclopedia of Toxicology.

[CR5] Borowik A, Wyszkowska J, Wyszkowski M (2017). Resistance of aerobic microorganisms and soil enzyme response to soil contamination with Ekodiesel Ultra fuel. Environmental Science and Pollution Research.

[CR6] Bronick CJ, Lal R (2005). Soil structure and management: A review. Geoderma.

[CR7] Burns RG, De Forest JL, Marxsen J, Sinsabaugh RL, Stromberger ME, Wallenstein MD, Weintraub MN, Zoppini A (2013). Soil enzymes in a changing environment: current knowledge and future directions. Soil Biology & Biochemistry.

[CR8] Chandurvelan R, Marsden ID, Gaw S, Glover CN (2013). Waterborne cadmium impacts immunocytotoxic and cytogenotoxic endpoints in green-lipped mussel, *Perna canaliculus*. Aquatic Toxicology.

[CR9] Cheynier V, Comte G, Davies KM, Lattanzio V, Martens S (2013). Plant phenolics: recent advances on their biosynthesis, genetics, and ecophysiology. Plant Physiology and Biochemistry.

[CR10] Daglia M (2012). Polyphenols as antimicrobial agents. Current Opinion in Biotechnology.

[CR11] De Leij F. A. A. M., Whipps J. M., Lynch J. M. (1994). The use of colony development for the characterization of bacterial communities in soil and on roots. Microbial Ecology.

[CR12] Derrien D, Marol C, Balesdent J (2004). The dynamics of neutral sugars in the rhizosphere of wheat: an approach by 13C pulse-labelling and GC/C/IRMS. Plant and Soil.

[CR13] Diaz S, Kattge J, Cornelissen JH, Wright IJ, Lavorel S, Dray S, Reu B, Kleyer M, Wirth C, Prentice IC, Garnier E, Bonisch G, Westoby M, Poorter H, Reich PB, Moles AT, Dickie J, Gillison AN, Zanne AE, Chave J, Wright SJ, Sheremet’ev SN, Jactel H, Baraloto C, Cerabolini B, Pierce S, Shipley B, Kirkup D, Casanoves F, Joswig JS, Gunther A, Falczuk V, Ruger N, Mahecha MD, Gorne LD (2016). The global spectrum of plant form and function. Nature.

[CR14] Ferlian O, Wirth C, Eisenhauer N (2017). Leaf and root C-to-N ratios are poor predictors of soil microbial biomass C and respiration across 32 tree species. Pedobiologia.

[CR15] Grienke U, Silke J, Tasdemir D (2014). Bioactive compounds from marine mussels and their effects on human health. Food Chemistry.

[CR16] Grigoras AG (2017). Catalase immobilization-a review. Biochemical Engineering Journal.

[CR17] Guangming L, Xuechen Z, Xiuping W, Hongbo, Jingsong Y, Xiangping W (2017). Soil enzymes as indicators of saline soil fertility under various soil amendments. Agriculture, Ecosystems and Environment.

[CR18] Harwood CS, Burchhardt G, Herrmann H, Fuchs G (1999). Anaerobic metabolism of aromatic compounds via the benzoyl-CoA pathway. FEMS Microbiology Reviews.

[CR19] IUSS Working Group WRB. (2014). World reference base for soil resources: international soil classification system for naming soils and creating legends for soil maps. Rome: FAO.

[CR20] Kaisoon O, Siriamornpun S, Weerapreeyakul N, Meeso N (2011). Phenolic compounds and anti - oxidant activities of edible flowers from Thailand. Journal of Functional Foods.

[CR21] Kristanti RA, Zubir MMFA, Hadibarata T (2016). Biotransformation studies of cresol red by *Absidia spinosa* M15. Journal of Environmental Management.

[CR22] Kucharski J, Tomkiel M, Baćmaga M, Borowik A, Wyszkowska J (2016). Enzyme activity and microorganisms diversity in soil contaminated with the Boreal 58 WG. Journal of Environmental Science and Health - Part B Pesticides, Food Contaminants, and Agricultural Wastes.

[CR23] Lebeis SL, Paredes HS, Lundberg DS, Breakfield N, Gehring J, McDonald M, Malfatti S, Glavina del Rio T, Jones CD, Tringe SG, Dangl JL (2015). Salicylic acid modulates colonization of the root microbiome by specific bacterial taxa. Science.

[CR24] Manzoni S, Moyano F, Kätterer T, Schimel J (2016). Modeling coupled enzymatic and solute transport controls on decomposition in drying soils. Soil Biology & Biochemistry.

[CR25] Marrot B, Barrios – Martinez A, Moulin P, Roche N (2006). Biodegradation of high phenol concentration by activated sludge in an immersed membrane bioreactor. Biochemical Engineering Journal.

[CR26] Mazzei L, Cianci M, Musiani F, Ciurli S (2016). Inactivation of urease by 1,4-benzoquinone: chemistry at the protein surface. Dalton Transactions.

[CR27] Mazzei L, Cianci M, Musiani F, Lente G, Palombo M, Ciurli S (2017). Inactivation of urease by catechol: kinetics and structure. Journal of Inorganic Biochemistry.

[CR28] Mikami D, Kurihara H, Ono M, Kim SM, Takahashi K (2016). Inhibition of algal bromophenols and their related phenols against glucose 6-phosphate dehydrogenase. Fitoterapia.

[CR29] Morinaga Y, Fuke C, Arao T, Miyazaki T (2004). Quantitative analysis of cresol and its metabolites in biological materials and distribution in rats after oral administration. Legal Medicine.

[CR30] Nguyen TTH, Li S, Li J, Liang T (2013). Micro-distribution and fixation of a rosin based micronized-copper preservative in poplar wood. International Biodeterioration & Biodegradation.

[CR31] OECD (2005). SIDS.: *O*-cresol (Screening Information Data Set - SIDs) http://www.inchem.org/documents/sids/sids/95487.pdf.

[CR32] Orwin K.H., Wardle D.A. (2004). New indices for quantifying the resistance and resilience of soil biota to exogenous disturbances. Soil Biology and Biochemistry.

[CR33] Paterson Eric, Sim Allan, Osborne Shona M., Murray Phil J. (2011). Long-term exclusion of plant-inputs to soil reduces the functional capacity of microbial communities to mineralise recalcitrant root-derived carbon sources. Soil Biology and Biochemistry.

[CR34] Patil JS, Anil AC (2015). Efficiency of copper and cupronickel substratum to resist development of diatom biofilms. International Biodeterioration & Biodegradation.

[CR35] Ren Y, Peng L, Zhao G, Wei C (2014). Degradation of m-cresol via the ortcleavage pathway by *Citrobacter farmeri* SC01. Biochemical Engineering Journal.

[CR36] Sanders JM, Bucher JR, Peckham JC, Kissling GE, Hejtmancik MR, Chhabra RS (2009). Carcinogenesis studies of cresols in rats and mice. Toxicology.

[CR37] Sarathchandra SU, Burch G, Cox NR (1997). Growth patterns of bacterial communities in the rhizoplane and rhizosphere of with clover (*Trifolium repens* L.) and perennial ryegrass (*Lolium perenne* L.) in long – term pasture. Applied Soil Ecology.

[CR38] Schimel Joshua, Becerra Caryl Ann, Blankinship Joseph (2017). Estimating decay dynamics for enzyme activities in soils from different ecosystems. Soil Biology and Biochemistry.

[CR39] Srisunont Chayarat, Babel Sandhya (2015). Uptake, release, and absorption of nutrients into the marine environment by the green mussel ( Perna viridis ). Marine Pollution Bulletin.

[CR40] Statsoft Inc. (2018). Data analysis software system. Version 12.0. Available at: http://www.statsoft.com.

[CR41] Steinauer K, Fischer FM, Roscherd C, Scheue S, Eisenhauera N (2017). Spatial plant resource acquisition traits explain plant community effects on soil microbial properties. Pedobiologia - Journal of Soil Ecology.

[CR42] Subbarao G. V., Nakahara K., Hurtado M. P., Ono H., Moreta D. E., Salcedo A. F., Yoshihashi A. T., Ishikawa T., Ishitani M., Ohnishi-Kameyama M., Yoshida M., Rondon M., Rao I. M., Lascano C. E., Berry W. L., Ito O. (2009). Evidence for biological nitrification inhibition in Brachiaria pastures. Proceedings of the National Academy of Sciences.

[CR43] Takishima K, Suga T, Mamiya G (1988). The structure of jack bean urease. The complete amino acid sequence, limited proteolysis and reactive cysteine residues. European Journal of Biochemistry.

[CR44] Uzer A, Ercag E, Parlar H, Apak R, Filik H (2006). Spectrophotometric determination of 4,6-dinitro-o-cresol (DNOC) in soil and lemon juice. Analytica Chimica Acta.

[CR45] Vaquero RMJ, Fernandez APA, Nadra MMC, Saad SAM (2010). Phenolic compound combinations on *Escherichia coli* viability in a meat system. Journal of Agricultural and Food Chemistry.

[CR46] Vecino X, Devesa-Rey R, Cruz JM, Moldes AB (2013). Evaluation of biosurfactant obtained from *Lactobacillus pentosus* as foaming agent in froth flotation. Journal of Environmental Management.

[CR47] Wyszkowska, J., Borowik, A., Kucharski, M., & Kucharski, J. (2013). Applicability of biochemical indices to quality assessment of soil pulluted with heavy metal. *Journal of Elementology*. 10.5601/jelem.2013.18.4.504.

[CR48] Wyszkowska, J., Boros-Lajszner, E., Lajszner, W., & Kucharski, J. (2017). Reaction of soil enzymes and spring barley to copper chloride and copper sulphate. *Environmental Earth Sciences, 76*. 10.1007/s12665-017-6742-2.

[CR49] Xiao ZP, Shi DH, Li HQ, Zhang LN, Xu C, Zhu HL (2007). Polyphenols based on isoflavones as inhibitors of *Helicobacter pylori* urease. Bioorganic & Medicinal Chemistry.

[CR50] Zaborowska M, Kucharski J, Wyszkowska J (2016). Biological activity of soil contaminated with cobalt, tin and molybdenum. Environmental Monitoring and Assessment.

[CR51] Zaborowska, M., Kucharski, J., & Wyszkowska, J. (2017). Brown algae and basalt meal in maintaining the activity of arylsulfatase of soil polluted with cadmium. *Water, Air, and Soil Pollution*. 10.1007/s11270-017-3449-7. 10.1007/s11270-017-3449-7 PMC5501898 28747806

[CR52] Zhu Yong, Li Tong, Fu Xiong, Abbasi Arshad Mehmood, Zheng Bisheng, Liu Rui Hai (2015). Phenolics content, antioxidant and antiproliferative activities of dehulled highland barley (Hordeum vulgare L.). Journal of Functional Foods.

[CR53] Zhu X, Wu X, Yao J, Wang F, Liu W, Luo Y, Jiang X (2018). Toxic effects of binary toxicants of cresol frother and Cu (II) on soil microorganisms. International Biodeterioration & Biodegradation.

